# Mochi-related vs other food-related foreign body airway obstruction: outcomes from the MOCHI registry

**DOI:** 10.1016/j.resplu.2026.101257

**Published:** 2026-02-09

**Authors:** Yutaka Igarashi, Tatsuya Norii, Hatsumi Nakanishi, Ryuta Nakae, Shoji Yokobori, MOCHI Investigators, Shoichi Yoshiike, Kosuke Shiroto, Tatsuho Kobayashi, Hiroko Kikuchi, Riko Wakisaka, Yosuke Homma, Masakazu Obayashi, Yasuo Shichinohe, Daiki Sunada, Ryu Sugimoto, Atsushi Koyama, Tomomi Iwashita, Masato Miyauchi, Tadashi Kaneko, Kazushige Inoue, Eiju Hasegawa, Nobuhiro Sato, Kiyoshi Matsuda, Jun-ichi Inoue, Takashi Tagami, Tomohiro Hattori, Toru Miike, Mayuko Koba, Kosuke Nakano, Naoki Tominaga, Eichi Narimatsu, Naofumi Bunya, Satoshi Yamanouchi, Hiroshi Takase, Tetsuya Matsuoka, Shota Nakao, Sung-Ho Kim, Toru Hifumi, Kazuhito Tamehiro, Yuji Tokuda, Mariko Sugita, Yoshihide Nakagawa, Hirotsugu Kaneshima, Taku Funakoshi, Ririko Kuwana, Kenta Ishii, Satoshi Takao, Sunao Yamauchi

**Affiliations:** 1 Aizawa Hospital, Japan; 2 Aizu Chuo Hospital, Japan; 3 Ashikaga Red Cross Hospital, Japan; 4 Chiba Kaihin Municipal Hospital, Japan; 5 Chutoen General Medical Center, Japan; 6 Hokkaido Medical Center, Japan; 7 Hyogo Prefectural Tamba Medical Center, Japan; 8 Iwaki City Medical Center, Japan; 9 Japanese Red Cross Society Nagano Hospital, Japan; 10 Kochi Medical School Hospital, Japan; 11 Mie University Hospital, Japan; 12 National Disaster Medical Center, Japan; 13 Niigata City General Hospital, Japan; 14 Nippon Medical School Musashi-Kosugi Hospital, Japan; 15 Otemachi Hospital, Japan; 16 Saga University Hospital, Japan; 17 Saitama City Hospital, Japan; 18 Sapporo Medical University Hospital, Japan; 19 Sendai City Hospital, Japan; 20 Senshu Trauma and Critical Care Center, Japan; 21 St. Luke’s International Hospital, Japan; 22 St. Mary’s Hospital, Japan; 23 Tokai University Hachioji Hospital, Japan; 24 Tokai University Hospital, Japan; 25 Tokyo Bay Urayasu Ichikawa Medical Center, Japan; 26 Toyohashi Municipal Hospital, Japan; 27 Unnan City Hospital, Japan; 28 Yuuai Medical Center, Japan; a Department of Emergency and Critical Care Medicine, Nippon Medical School, Tokyo, Japan; b Department of Emergency Medicine, University of New Mexico, Albuquerque, USA; c Department of Traumatology and Acute Critical Medicine, Osaka University Graduate School of Medicine, Osaka, Japan; d Faculty of Medicine, Nippon Medical School, Tokyo, Japan

**Keywords:** Airway obstruction, Foreign bodies, Asphyxia, Cardiac arrest, Mochi

## Abstract

**Background:**

Mochi (Japanese rice cake) is frequently implicated in severe foreign body airway obstruction (FBAO) due to its distinctive viscoelasticity and adhesive properties. However, it remains unclear whether mochi-related FBAO is associated with worse outcomes.

**Methods:**

In this nationwide prospective multicenter observational study (MOCHI registry), we analyzed patients with food-related FBAO transported to hospitals. The exposure was mochi-related FBAO and the comparator was other food-related FBAO. The primary outcome was 30-day survival; secondary outcome included favorable neurological outcome (Cerebral Performance Category [CPC] 1–2). We employed inverse probability of treatment weighting (IPTW) based on a propensity score model including age, sex, baseline functional status, comorbidities, event setting, witness status, and the New Year period.

**Results:**

Of 409 patients assessed for eligibility in the MOCHI registry, 399 were included in the final analysis; 63 (15.8%) presented with mochi-related FBAO. Mochi cases were more often ADL-independent (44/61 [72.1%] vs 128/329 [38.9%]) and had fewer comorbidities (e.g., dementia: 10/63 [15.9%] vs 116/336 [34.5%]; prior stroke: 5/63 [7.9%] vs 74/336 [22.0%]) and clustered at home during the New Year period. In the IPTW analysis, mochi was not significantly associated with 30-day survival (IPTW-weighted OR, 0.93; 95% CI, 0.41–2.11) or favorable neurological outcomes (IPTW-weighted OR, 0.87; 95% CI, 0.30–2.49).

**Conclusions:**

Mochi was not statistically significantly associated with 30-day outcomes after adjustment, but estimates were imprecise. Mochi-related events occurred disproportionately among relatively healthier, functionally independent individuals and occurred predominantly at home during seasonal consumption, supporting targeted seasonal prevention and public education in home settings.

## Introduction

Foreign body airway obstruction (FBAO) represents a critical and treatable public health challenge characterized by the rapid onset of hypoxia due to upper airway occlusion^1^. Without immediate intervention, FBAO can rapidly progress to cardiac arrest. In the United States, over 5000 fatal FBAO episodes are reported annually^2^, while in Japan, FBAO due to food accounts for approximately 4000 deaths each year^3^, ^4^.

A wide variety of foods can cause airway obstruction, including meat, bread, rice, vegetables, and fruits^5^, ^6^, ^7^, ^8^, ^9^. In Japan, “mochi (traditional rice cake)” is a major cause of seasonal choking, especially during the New Year period^6^, ^7^, ^10^, ^11^. Its high viscoelasticity and sticky texture make removal difficult^10^. Culturally, mochi is a staple food consumed to celebrate the New Year; consequently, a substantial proportion of mochi-related FBAO incidents occur during the first three days of January^12^. Furthermore, mochi products including mochi-based desserts are increasingly available outside Japan^13^. Because highly viscoelastic and adhesive goods can cause time-critical FBAO in any community, our findings may inform prevention messaging an early response beyond Japan.

Although mochi is considered a high-risk food due to its physical properties, it remains unclear whether mochi itself is associated with worse clinical outcomes compared to other food items among patients transported to hospitals with food-related FBAO. Previous studies have often lacked rigorous adjustment for confounding factors such as patient frailty, event location, and seasonality^12^. Therefore, we aimed to compare baseline characteristics and 30-day survival and neurological outcomes between patients with FBAO caused by mochi and those with FBAO caused by other foods in a nationwide prospective multicenter registry, prespecifying the hypothesis that mochi-related FBAO would be associated with worse outcomes.

## Methods

### Study design and setting

This was a prospective, nationwide, multicenter observational study in Japan using the Multicenter Observational CHoking Investigation (MOCHI) registry^14^. We included all patients with FBAO who were transported to the participating hospitals from April 2020 to March 2023. We defined FBAO as acute mechanical airway obstruction caused by foreign body in the airway. Patients were excluded if 30-day outcomes were missing or if the foreign body was a non-food object. The study protocol was approved by the Institutional Review Board of Nippon Medical School Hospital (Approval No. B-2019-019) and the ethics committees of all participating hospitals.

### Data collection and variables

The primary exposure was airway obstruction caused by mochi. The comparator group comprised all other food-related obstructions, including cases in which the specific food type was unspecified. We collected baseline data including age, sex, activities of daily living (ADL) status (independent vs. dependent), pre-event comorbidities (cerebral infarction, dementia, schizophrenia, and history of choking), event location (private vs. public), witness status (witnessed vs. unwitnessed), and bystander intervention (any attempt to relieve obstruction before emergency medical services arrival). Out-of-hospital cardiac arrest (OHCA) was defined as cardiac arrest occurring before hospital arrival, regardless of whether cardiopulmonary resuscitation was initiated by bystanders or emergency medical services (EMS). Events during the New Year’s period (January 1–3) were also identified. Patients were classified as independent in basic ADLs if they were independent in bathing, dressing, toileting, transferring, continence, and feeding^15^. Because feeding is a component of the basic ADL definition, we did not additionally adjust for eating function to avoid potential multicollinearity. These comorbidities were prespecified as clinically relevant factors related to dysphagia and cognitive/behavioral factors and were included as potential confounders^16^, ^17^, ^18^, ^19^, ^20^.

At each participating site, treating physicians in the emergency department obtained event details (including food type) from witnesses and EMS personnel using a structured interview form as specified in the MOCHI protocol; interviews were conducted as early as possible after hospital arrival, in person when feasible. To ensure data quality and inter-site consistency, all investigators used a standardized data dictionary and case-report form with predefined variable definitions.

### Outcome measures

Considering the high prevalence of pre-existing disabilities and advanced age in our study cohort, we selected 30-day survival as the primary outcome to avoid the floor effect of functional scales. Secondary outcomes included 30-day favorable neurological outcomes, defined as a Cerebral Performance Category (CPC) of 1 or 2 and an expanded neurological outcome (CPC 1–3)^21^. The CPC 1–3 category was included to capture meaningful recovery in patients with pre-event functional limitations who may not meet CPC 1 or 2 despite returning to baseline.

### Statistical analysis

As age and airway obstruction time were non-normally distributed, they are presented as median [interquartile range, IQR] and compared between groups using the Mann-Whitney *U* test. Categorical variables are presented as numbers (percentages) and compared using the Chi-square test or Fisher's exact test as appropriate.

To address missing data in baseline covariates, we performed multiple imputation by chained equations (MICE) assuming the data were missing-at-random, generating 20 imputed datasets.

To minimize confounding bias, we employed an inverse probability of treatment weighting (IPTW) approach. The propensity score for the obstruction being caused by mochi was estimated in each imputed dataset using a logistic regression model including age, sex, ADL status, pre-event comorbidities, location of the event, witness status, and whether the incident occurred during the New Year period (January 1–3). Unstabilized IPTW weights were truncated at the 99th percentile to mitigate the influence of extreme weights. Covariate balance was assessed using absolute standardized mean differences (SMD), with an SMD < 0.1 considered indicative of negligible imbalance.

We compared outcomes between the groups using weighted logistic regression models. To account for the clustering of patients within hospitals, we used robust standard errors clustered by institution. Results from the 20 imputed datasets were pooled using Rubin’s rules. Because IPTW was used to balance baseline characteristics, outcome models were not further adjusted for covariates. Results are presented as IPTW-weighted odds ratios (ORs) with 95% confidence intervals (CI).

We conducted three prespecified sensitivity analyses: (1) a complete-case IPTW analysis without multiple imputation; (2) an alternative confounder specification replacing basic ADL with eating function in the propensity score model; and (3) restriction of the comparator group to “known other foods only” by excluding cases with an unknown food type from other foods group. All sensitivity analyses used the same IPTW framework and outcome modeling approach as the primary analysis.

For descriptive purposes, unadjusted 30-day survival curves were estimated using the Kaplan-Meier method and compared using the log-rank test. IPTW-weighted Kaplan–Meier curves were additionally plotted descriptively.

For an exploratory causal mediation analysis, we used OHCA as a clinically relevant intermediate event between exposure (mochi) and 30-day survival to assess whether progression to cardiac arrest could account for any observed association; effects were estimated on the risk-difference scale with 1000 bootstrap samples, adjusting for the same baseline covariates as the propensity score model and interpreted as hypothesis-generating. Mediation estimates are reported on the risk-difference scale.

All statistical analyses were performed using R (The R Foundation for Statistical Computing, Vienna, Austria). A two-sided *P*-value of <0.05 was considered statistically significant.

## Results

**Fig. 1:**
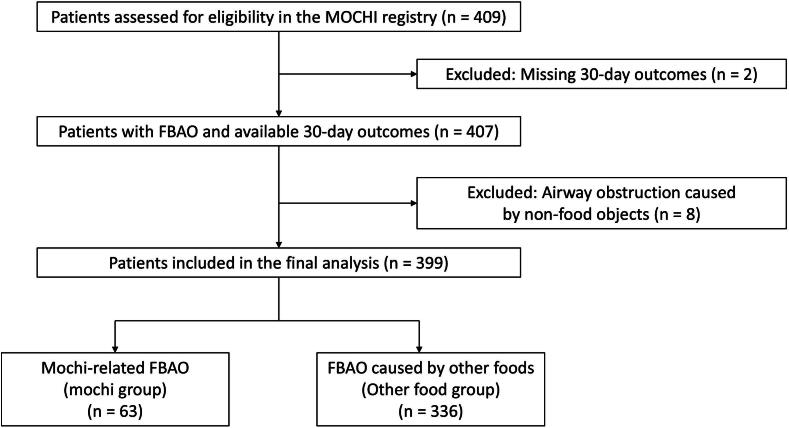
**Study flowchart showing patient selection and group assignment based on the MOCHI registry**.

Of 409 patients assessed for eligibility in the MOCHI registry, 399 patients were included in the final analysis (Fig. 1), of whom 63 patients (15.8%) had airway obstruction caused by mochi. The distribution of food types (with no missing data and therefore not imputed) is shown in Table 1. Compared with the other foods group, the mochi group had a similar median age (82.0 [77.0–86.5] vs 81.0 [73.0–88.0] years; *p* = 0.557) but more males (43/63 [68.3%] vs. 172/336 [51.2%]; *p* = 0.018) and a nearly two-fold higher proportion of patients who were independent in ADLs (44/61 [72.1%] vs. 128/329 [38.9%]; *p* < 0.001). The mochi group had a lower prevalence of cerebral infarction (5/63 [7.9%] vs 74/336 [22.0%]; *p* = 0.009) and dementia (10/63 [15.9%] vs 116/336 [34.5%]; *p* = 0.006). Events in the mochi group more frequently occurred in private locations (60/63 [95.2%] vs 283/336 [83.2%]; *p* = 0.012), particularly at home (57/63 [90.5%] vs 161/336 [47.9%]; *p* < 0.001), and less frequently in nursing homes (1/63 [1.6%] vs 84/336 [25.0%]; *p* < 0.001). Mochi-related events were more common on January 1 (12/63 [19.0%] vs 20/336 [6.0%]; *p* = 0.001) and during the New Year period (January 1–3) (20/63 [31.7%] vs 44/336 [13.1%]; *p* < 0.001) (Table 2). Bystander interventions and OHCA-related event characteristics are summarized in Table S1. A total of 36 patients (9.0%) had ≥1 missing covariate and were imputed (Table S2).

**Table 1:** Distribution of food types causing foreign body airway obstruction.

**Food type**	**Count (%)**
Rice	86 (21.6%)
Rice cake (mochi)	63 (15.8%)
Meat	61 (15.3%)
Bread	32 (8.0%)
Vegetable	32 (8.0%)
Fruit	18 (4.5%)
Noodle	14 (3.5%)
Fish	13 (3.3%)
Jelly	12 (3.0%)
Sushi	10 (2.5%)
Potato	5 (1.3%)
Others	84 (21.1%)
Unknown	26 (6.5%)

**Table 2:** Patient characteristics of patients with food-related foreign body airway obstruction by food type (mochi vs other foods).

**Variable**	**Total** **(*N* = 399)**	**Mochi** **(*n* = 63)**	**Other foods** **(*n* = 336)**	**Absolute difference** **[95% CI]**	***P* value**
Age	82.0 [73.0–87.5]	82.0 [77.0–86.5]	81.0 [73.0–88.0]	1.0 [−1.0, 3.0]	0.557
Sex (male)	215/399 (53.9%)	43/63 (68.3%)	172/336 (51.2%)	17.1% [4.4, 29.7]	0.018
ADL independent	172/390 (44.1%)	44/61 (72.1%)	128/329 (38.9%)	33.2% [20.8, 45.6]	<0.001
**Past medical history**					
Cerebral infarction	79/399 (19.8%)	5/63 (7.9%)	74/336 (22.0%)		0.009
Dementia	126/399 (31.6%)	10/63 (15.9%)	116/336 (34.5%)	−18.7% [−29.0, −8.3]	0.006
Schizophrenia	19/399 (4.8%)	1/63 (1.6%)	18/336 (5.4%)		0.332
Choking	8/399 (2.0%)	0/63 (0.0%)	8/336 (2.4%)		0.366
Private location†	339/399 (85.0%)	60/63 (95.2%)	283/336 (83.2%)	12.2% [5.6, 18.8]	0.012
**Location**					
Day care center	12/399 (3.0%)	0 (0.0%)	12 (3.6%)		0.227
Group home	36/399 (9.0%)	2/63 (3.2%)	34 (10.1%)		0.093
Home	218/399 (54.6%)	57/63 (90.5%)	161 (47.9%)	42.6% [33.6, 51.6]	<0.001
Nursing home	85/399 (21.3%)	1/63 (1.6%)	84 (25.0%)		<0.001
Others	60/399 (15.0%)	3/63 (4.8%)	57 (17.0%)		0.172
1 January	32/399 (8.0%)	12/63 (19.0%)	20/336 (6.0%)	13.1% [3.1, 23.1]	0.001
1–3 January	64/399 (16.0%)	20/63 (31.7%)	44/336 (13.1%)	18.7% [6.6, 30.7]	<0.001
Witness	347 (87.0%)	59/63 (93.7%)	288/336 (85.7%)	8.5% [1.4, 15.6]	0.103
Bystander interventions	189/371 (50.9%)	22/53 (41.5%)	167/318 (52.5%)	−11.0% [−25.4, 3.3]	0.182
Airway obstruction time (min), median [IQR]	16.0 [6.0, 35.0]	15.0 [9.0, 25.0]	17.0 [5.0, 35.0]	−2.00 [−6.00, 2.00]	0.364

ADL, activities of daily living; CI, confidence interval; IQR, interquartile range. Percentages are calculated using non-missing denominators.

For variables with cell counts of 5 or fewer, Fisher’s exact test was used, and absolute differences and 95% CIs were not reported due to the instability of estimates.

† Private location included group home, home, and nursing home.

**Supplementary Fig. S1:**
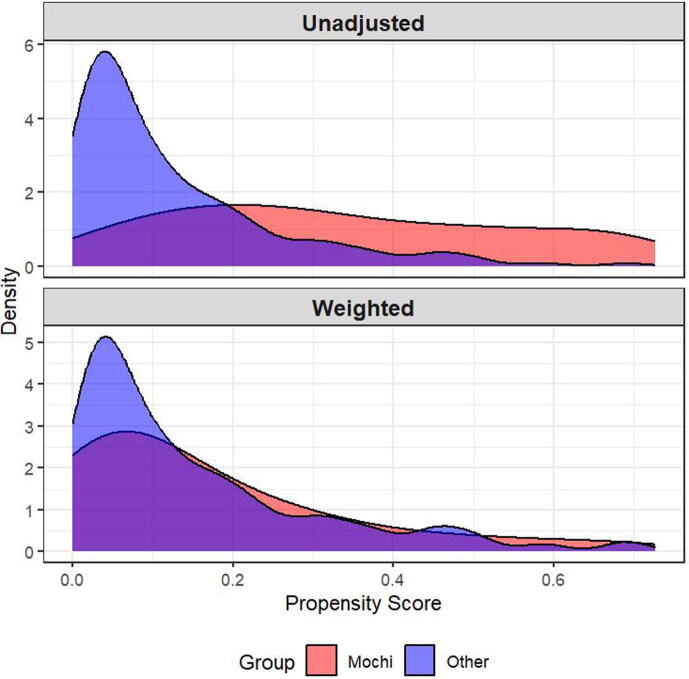
Propensity score distribution before and after IPTW.

**Supplementary Fig. S2:**
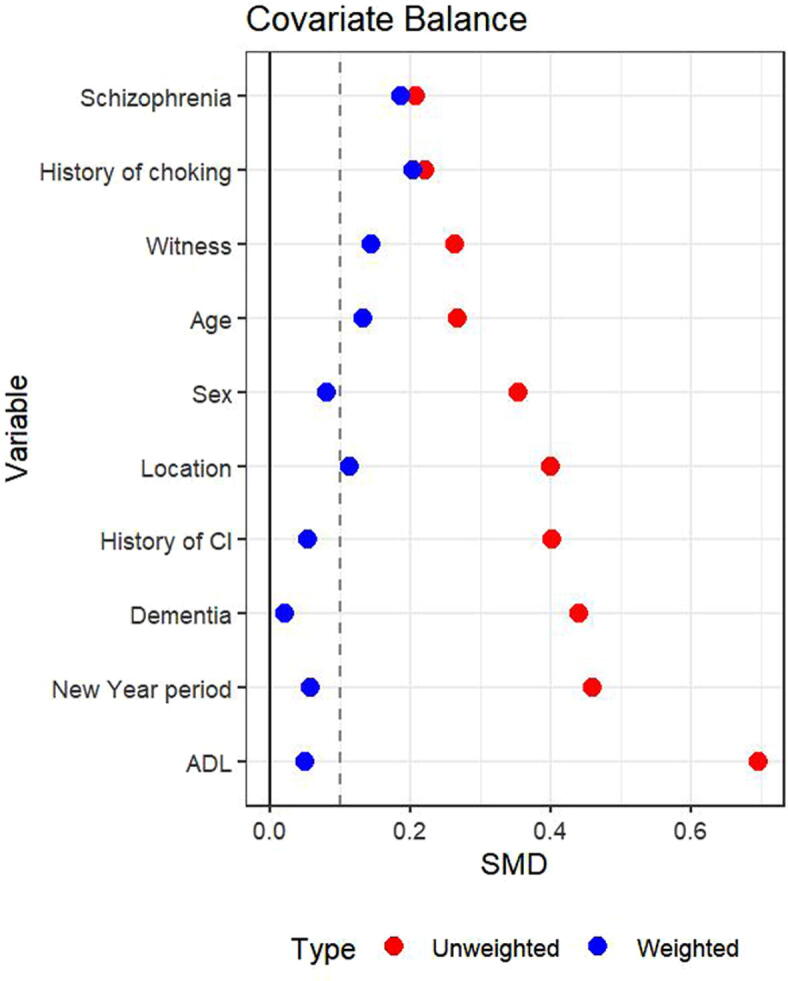
Covariate balance before and after IPTW.

After IPTW adjustment, covariate balance was substantially improved (Fig. S1). Weight diagnostics for the IPTW procedure are provided in Table S3. Absolute SMDs for all covariates with large initial imbalances were reduced, although slight residual imbalances remained for schizophrenia, history of choking, age, witness present, and private location (Fig. S2).

In the raw cohort, 30-day survival was 32/63 (50.8%) in the mochi group and 155/336 (46.1%) in the other foods group (*p* = 0.496). Favorable neurological outcome occurred in 16/63 (25.4%) vs 78/336 (23.2%), and CPC 1–3 outcomes occurred in 22/63 (34.9%) vs 132/336 (39.3%). Similarly, stratified analyses revealed no significant differences in survival rates between the two groups regardless of OHCA status (Table 3). After IPTW adjustment, mochi-related FBAO was not significantly associated with 30-day survival (IPTW-weighted OR, 0.93; 95% confidence interval [CI], 0.41–2.11), favorable neurological outcome (IPTW-weighted OR, 0.87; 95% CI, 0.30–2.49), or CPC 1–3 outcome (IPTW-weighted OR, 0.75; 95% CI, 0.30–1.86) (Table 4). Sensitivity analyses yielded findings consistent with the primary analysis (Table S4).

**Table 3:** Thirty-day survival by food type (mochi vs other foods), overall and stratified by out-of-hospital cardiac arrest.

	**Total**	**Mochi**	**Other foods**	***P* value**
Overall	187/399 (46.9%)	32/63 (50.8%)	155/336 (46.1%)	0.496
In OHCA case	50/213 (23.5%)	13/39 (33.3%)	37/174 (21.3%)	0.108
In non-OHCA case	137/186 (73.7%)	19/24 (79.2%)	118/162 (72.8%)	0.511

OHCA, out-of-hospital cardiac arrest.

**Table 4:** Clinical outcomes comparing mochi-related versus other food-related FBAO: unadjusted and IPTW-weighted effect estimates.

	**Mochi** **(*n* = 63)**	**Other foods** **(*n* = 336)**	**Unadjusted OR** **(95% CI)**	**IPTW-weighted OR** **(95% CI)**
**Primary outcomes**				
Survival at 30 days	32 (50.8%)	155 (46.1%)	1.22 (0.71–2.10)	0.93 (0.41–2.11)
**Secondary outcomes**				
Favorable neurological outcomes (CPC 1 or 2)	16 (25.4%)	78 (23.2%)	1.11 (0.59–2.07)	0.87 (0.30–2.49)
Neurological outcomes with CPC 1–3	22 (34.9%)	132 (39.3%)	0.83 (0.47–1.46)	0.75 (0.30–1.86)

IPTW-weighted ORs were estimated using weighted logistic regression with institution-clustered robust standard errors. Covariates were balanced through IPTW based on the propensity score model including age, sex, ADL, comorbidities, location, witness status, and the New Year period.

CI, confidence interval; CPC, Cerebral Performance Category; IQR, interquartile range; OR, odds ratio.

**Fig. 2:**
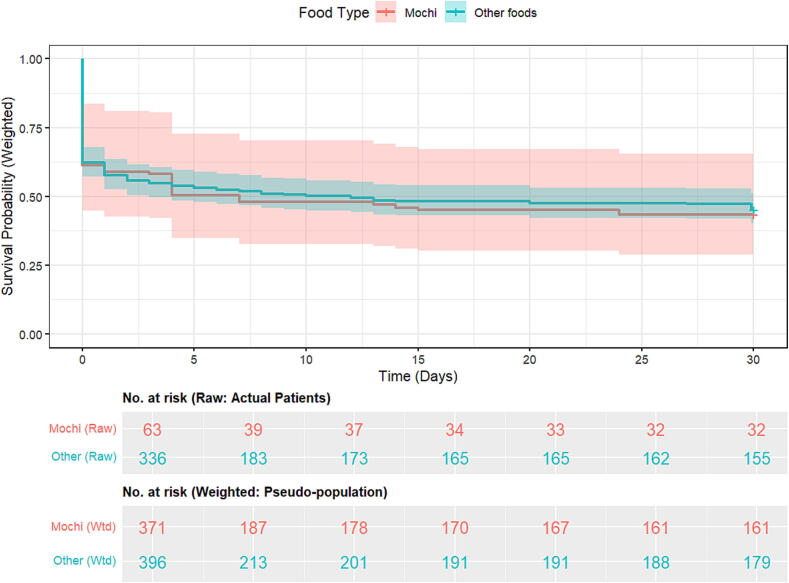
**IPTW-weighted Kaplan–Meier curves for 30-day survival comparing mochi-related and other food-related FBAO were plotted descriptively (shaded areas indicate 95% confidence intervals). Numbers at risk are shown for the raw cohort (upper) and the IPTW-weighted pseudo-population (lower)**.

The IPTW-weighted Kaplan–Meier survival curves for 30-day survival are shown in Fig. 2. In the unweighted Kaplan–Meier analysis of the raw cohort, there was no significant difference in 30-day survival between the mochi and other foods groups (log-rank test, *p* = 0.39).

Causal mediation analysis was performed to decompose the total effect of airway obstruction caused by mochi on 30-day survival. The total effect in this exploratory model was 0.12 (*p* = 0.092). The average direct effect (ADE) of airway obstruction caused by mochi on survival was statistically significant (Estimate, 0.15; *p* = 0.018). Conversely, the average causal mediation effect (ACME) through OHCA was small and not statistically significant (Estimate, −0.03; *p* = 0.476). These findings should be interpreted cautiously given strong identification assumptions and potential model instability.

## Discussion

In this nationwide prospective multicenter registry of patients transported to hospital with life-threatening, food-related FBAO, we did not detect an association between mochi-related FBAO and worse 30-day survival or neurological outcomes after IPTW adjustment. However, the effect estimates were imprecise with wide confidence intervals, and clinically important differences cannot be excluded.

The mochi group was approximately twice as likely to have independent ADL and had a lower comorbidity rate, suggesting a selection process based on functional status^9^, ^22^. Because mochi is widely recognized as a high-risk food for choking, it is likely that individuals with impaired swallowing functions or severe frailty either avoid mochi consumption or have their intake restricted by caregivers. Consequently, mochi-related FBAO predominantly involves a relatively healthier population with higher biological reserves, representing a more “accidental” pattern of choking. In contrast, FBAO caused by other common foods may more frequently involve frailer patients, where the event reflects an underlying vulnerability in swallowing and protective reflexes, representing a more ”pathological” pattern.

After accounting for measured confounders via IPTW, mochi-related FBAO was not statistically significantly associated with inferior clinical outcomes compared with other food items. However, the confidence intervals were wide and the modest number of mochi cases limited precision, such that clinically important differences cannot be ruled out. Therefore, these findings should be interpreted as inconclusive rather than as evidence that mochi has no effect on prognosis. We speculate that although mochi can be difficult to dislodge because of its viscoelasticity and adhesiveness, it may sometimes remain as a cohesive mass that could facilitate “en bloc” removal by trained rescuers using Magill forceps. Reports showing better outcomes among OHCA patients who underwent prehospital foreign body removal with Magill forceps may be relevant in this context, although causality cannot be inferred^23^.

Our exploratory mediation analysis suggested no statistically significant indirect effect via OHCA. While the ADE was positive, these results were not fully concordant with the primary IPTW analysis and require strong assumptions; therefore, they should be considered strictly hypothesis-generating. The positive ADE is consistent with the hypothesis that, if OHCA is avoided, mochi-related cases may have relatively favorable survival; however, this interpretation remains speculative.

These implications are relevant not only to mochi in Japan but also to other settings where similar high-risk, sticky foods are consumed, underscoring the universal importance of rapid recognition and immediate bystander maneuvers.

This study has several limitations. First, as an observational analysis, residual confounding may persist despite adjustment using IPTW, including the influence of unmeasured confounders. For instance, swallowing function may differ significantly even among patients with the same ADL status. Second, we lacked detailed information on the physical characteristics of the foreign body and the precise location and extent of obstruction, which may modify both the likelihood of OHCA and the success of removal maneuvers. Third, the comparator group was heterogeneous and included multiple food types with differing physical properties; grouping these diverse items together may have obscured food-specific differences. Fourth, the modest number of mochi cases limited precision, and the study may have been underpowered to detect modest but clinically meaningful differences (risk of Type II error). Finally, our registry includes only patients transported to participating hospitals, which may overrepresent severe cases. However, because prehospital termination of resuscitation (TOR) is generally not permitted in Japan^24^, non-transport after EMS contact and the absence of EMS-initiated TOR are uncommon; nevertheless, this may limit generalizability of our findings to all community FBAO events.

## Conclusion

In this nationwide prospective multicenter registry of patients with food-related FBAO, mochi-related FBAO was not statistically significantly associated with 30-day survival or neurological outcomes after confounder adjustment; however, estimates were imprecise and clinically important differences cannot be excluded. Mochi-related events occurred disproportionately among relatively healthier, functionally independent individuals and occurred predominantly at home during the New Year period. These findings support targeted seasonal prevention strategies and public education emphasizing food modification and prompt first-aid responses in home settings.

## Declaration of generative AI and AI-assisted technologies in the manuscript preparation process

During the preparation of this work, the authors used ChatGPT (OpenAI; GPT-5.2) and Gemini (Google) in order to improve the clarity and readability of the English text and to support editorial revision. After using these tools, the authors reviewed and edited the content as needed and take full responsibility for the content of the published article.

## CRediT authorship contribution statement

**Yutaka Igarashi:** Writing – original draft, Project administration, Methodology, Investigation, Funding acquisition, Formal analysis, Data curation, Conceptualization. **Tatsuya Norii:** Writing – review & editing, Supervision, Project administration, Methodology, Investigation, Funding acquisition, Conceptualization. **Hatsumi Nakanishi:** Data curation. **Ryuta Nakae:** Writing – review & editing. **Shoji Yokobori:** Writing – review & editing, Supervision, Funding acquisition.

## Funding

This work was supported by the Japanese Association for Acute Medicine, the Ministry of Education, Culture, Sports, Science and Technology (MEXT), Japan (Program for Developing Advanced Medical Human Resources) and the Taiju Life Social Welfare Foundation.

## Declaration of competing interest

The authors declare that they have no known competing financial interests.

## Appendix A Supplementary material

MOCHI Investigators
