# Supplementary material for: Mochi-related vs other food-related foreign body airway obstruction: outcomes from the MOCHI registry
Source: Resusc Plus. 2026 Feb 9;28:101257. doi: 10.1016/j.resplu.2026.101257 (PMC13404280; doi:10.1016/j.resplu.2026.101257)
Supplement: MOCHI Investigators [file mmc1.docx]

**Appendix 1. MOCHI Investigators (in alphabetical order by institution)**

Aizawa Hospital: Shoichi Yoshiike, MD; Kosuke Shiroto, MD
Aizu Chuo Hospital: Tatsuho Kobayashi, MD
Ashikaga Red Cross Hospital: Hiroko Kikuchi, MD; Riko Wakisaka, MD
Chiba Kaihin Municipal Hospital: Yosuke Homma, MD
Chutoen General Medical Center: Masakazu Obayashi, MD
Hokkaido Medical Center: Yasuo Shichinohe, MD; Daiki Sunada, MD
Hyogo Prefectural Tamba Medical Center: Ryu Sugimoto, MD
Iwaki City Medical Center: Atsushi Koyama, MD
Japanese Red Cross Society Nagano Hospital: Tomomi Iwashita, MD
Kochi Medical School Hospital: Masato Miyauchi, MD
Mie University Hospital: Tadashi Kaneko, MD
National Disaster Medical Center: Kazushige Inoue, MD; Eiju Hasegawa, MD
Niigata City General Hospital: Nobuhiro Sato, MD
Nippon Medical School Musashi-Kosugi Hospital: Kiyoshi Matsuda, MD; Jun-ichi Inoue, MD; Takashi Tagami, MD
Otemachi Hospital: Tomohiro Hattori, MD
Saga University Hospital: Toru Miike, MD; Mayuko Koba, MD
Saitama City Hospital: Kosuke Nakano, MD; Naoki Tominaga, MD
Sapporo Medical University Hospital: Eichi Narimatsu, MD; Naofumi Bunya, MD
Sendai City Hospital: Satoshi Yamanouchi, MD; Hiroshi Takase, MD
Senshu Trauma and Critical Care Center: Tetsuya Matsuoka, MD; Shota Nakao, MD; Sung-Ho Kim, MD
St. Luke’s International Hospital: Toru Hifumi, MD
St. Mary’s Hospital: Kazuhito Tamehiro, MD; Yuji Tokuda, MD
Tokai University Hachioji Hospital: Mariko Sugita, MD
Tokai University Hospital: Yoshihide Nakagawa, MD; Hirotsugu Kaneshima, MD
Tokyo Bay Urayasu Ichikawa Medical Center: Taku Funakoshi, MD; Ririko Kuwana, MD
Toyohashi Municipal Hospital: Kenta Ishii, MD
Unnan City Hospital: Satoshi Takao, MD
Yuuai Medical Center: Sunao Yamauchi, MD
