# Supplementary material for: Mochi-related vs other food-related foreign body airway obstruction: outcomes from the MOCHI registry
Source: Resusc Plus. 2026 Feb 9;28:101257. doi: 10.1016/j.resplu.2026.101257 (PMC13404280; doi:10.1016/j.resplu.2026.101257)
Supplement: Supplementary Table S1 [file mmc2.docx]

**Table S1. Bystander interventions and OHCA-related event characteristics in mochi-related versus other food-related FBAO.**

| **Variable** | **Total** (N = 399) | **Mochi**  (n = 63) | **Other foods**  (n = 336) | **P value** |
| --- | --- | --- | --- | --- |
| **Bystander intervention** | 189/371 (50.9%) | 22/53 (41.5%) | 167/318 (52.5%) | 0.182 |
| **Procedures** |  |  |  |  |
| Abdominal thrusts | 23/371 (6.2%) | 4/53 (7.5%) | 19/318 (6.0%) | 0.725 |
| Back blows | 75/371 (20.2%) | 9/53 (17.0%) | 66/318 (20.8%) | 0.655 |
| Remove with hand | 33/371 (8.9%) | 3/53 (5.7%) | 30/318 (9.4%) | 0.578 |
| Suction | 38/371 (10.2%) | 2/53 (3.8%) | 36/318 (11.3%) | 0.150 |
| Vacuum cleaner | 1/371 (0.3%) | 1/53 (1.9%) | 0/318 (0.0%) | 0.143 |
| **OHCA** | 213/399 (53.4%) | 39/63 (61.9%) | 174/336 (51.8%) | 0.180 |
| **Bystander CPR** | 169/213 (79.3%) | 27/39 (69.2%) | 142/174 (81.6%) | 0.117 |
| **ROSC** | 176/213 (82.6%) | 36/39 (92.3%) | 140/174 (80.5%) | 0.125 |
| On the scene | 53/213 (24.9%) | 7/39 (17.9%) | 46/174 (26.4%) | 0.354 |
| En route | 54/213 (25.4%) | 14/39 (35.9%) | 40/174 (23.0%) | 0.134 |

CPR, cardiopulmonary resuscitation; FBAO, foreign body airway obstruction; OHCA, out-of-hospital cardiac arrest; ROSC, return of spontaneous circulation.

**Table S2. Summary of missing data for covariates included in the propensity score model.**

| **Covariates** | **Missing count** (%) |
| --- | --- |
| Bystander removal attempts | 28 (7.0%) |
| ADL status | 9 (2.3%) |
| Age | 0 (0%) |
| Sex | 0 (0%) |
| Pre-event comorbidities | 0 (0%) |
| Witness status | 0 (0%) |
| Events during New Year’s period | 0 (0%) |

**Table S3. Propensity score weight diagnostics**

| **Diagnostic metric** | **Value** |
| --- | --- |
| Weighting method | Unstabilized Inverse Probability of Treatment Weighting |
| Trimming Strategy | Truncated at the 99th percentile |
| Total Sample Size | 399 |
| Mean Weight (Min /Median/Max) | 1.00/1.11/24.66 |
| Mean Cutoff for Trimming (99th percentile) | 24.66 |
| Percentage of Cases Trimmed | 1.00% |
| Effective Sample Size | 105.7 |

**Table S4. Sensitivity analyses for the association between mochi-related FBAO and 30-day outcomes (IPTW-weighted analyses)**

|  | **Complete-case analysis** | **ADL replaced with eating function** | **Known other foods only** |
| --- | --- | --- | --- |
| **Primary outcomes** |  |  |  |
| Survival at 30 days | 0.90 (0.38–2.11) | 0.93 (0.41–2.11) | 0.83 (0.36–1.90) |
| **Secondary outcomes** |  |  |  |
| Favorable neurological outcomes (CPC 1 or 2) | 0.92 (0.32–2.64) | 0.87 (0.30–2.49) | 0.87 (0.32–2.39) |
| Neurological outcomes with CPC 1–3 | 0.75 (0.31–1.80) | 0.75 (0.30–1.86) | 0.69 (0.29–1.62) |

Values are IPTW-weighted odds ratios (ORs) with 95% confidence intervals (CI) for mochi-related vs other food-related FBAO. Complete-case analysis excluded patients with missing covariates/outcomes required for the model. ADL to eating function indicates the propensity score model replaced basic ADL with eating function as a covariate. Excluding unknown foods restricted the comparator to known other foods only, excluding others/unknown food types. ADL, activity of daily living
